# Mounier-Kuhn Syndrome in an Elderly Female with Pulmonary Fibrosis

**DOI:** 10.1155/2016/8708251

**Published:** 2016-08-15

**Authors:** Panagiotis Boglou, Nikolaos Papanas, Anastasia Oikonomou, Stamatia Bakali, Paschalis Steiropoulos

**Affiliations:** ^1^Department of Pneumonology, Medical School, Democritus University of Thrace, 68100 Alexandroupolis, Greece; ^2^Second Department of Internal Medicine, Medical School, Democritus University of Thrace, 68100 Alexandroupolis, Greece; ^3^Department of Radiology, Medical School, Democritus University of Thrace, 68100 Alexandroupolis, Greece; ^4^Department of Microbiology, Medical School, Democritus University of Thrace, 68100 Alexandroupolis, Greece

## Abstract

Mounier-Kuhn syndrome (MKS), or tracheobronchomegaly, is a rare clinical and radiologic condition characterized by pronounced tracheobronchial dilation and recurrent lower respiratory tract infections. Tracheobronchomegaly presents when the defect extends to the central bronchi. MKS can be diagnosed in adult women when the transverse and sagittal diameters of the trachea, right mainstem bronchus, and left mainstem bronchus exceed 21, 23, 19.8, and 17.4 mm, respectively. Its diagnosis is based on chest radiograph and chest computed tomography (CT). Patients, usually middle-aged men, may be asymptomatic or present with clinical manifestations ranging from minimal symptoms with preserved lung function to severe respiratory failure. Pulmonary function tests (PFTs) typically reveal a restrictive pattern. This report presents an elderly woman with previously diagnosed pulmonary fibrosis with symptoms of increased sputum production and haemoptysis. High-resolution chest CT showed tracheal and main stem bronchi dilatation along with bronchial diverticulosis. PFTs indicated a restrictive pattern characteristic of the underlying pulmonary fibrosis. The patient is the oldest, referred to the female gender, at presentation of MKS hitherto reported. This case highlights the need to include MKS in the differential diagnosis of recurrent lower respiratory tract infections, even in older subjects.

## 1. Introduction

Mounier-Kuhn syndrome (MKS) is an infrequent congenital syndrome, whose hallmark is airway enlargement. On histological examination, absence or atrophy of the elastic fibres within the tracheal wall is typically found [1–7]. This condition results in airway dilatation in the trachea and bronchi. Tracheal diverticula may also occur, mainly in the posterior trachea [8]. The typical features of this entity were first described by Mounier-Kuhn in 1932 [9].

Patients usually complain of recurrent respiratory tract infections along with various functional perturbations: from minimal involvement with preservation of lung function, through severe disease with bronchiectasis to overt respiratory failure [1–7, 10]. Patients are typically middle-aged males [2–4]. Its diagnosis is based on chest radiograph and chest computed tomography (CT). MKS is frequently overlooked, but should be considered in the investigation of recurrent lower respiratory tract infections [1–9].

This report presents a female patient, the oldest hitherto described, with MKS who had been previously diagnosed with pulmonary fibrosis.

## 2. Case Presentation

An 83-year-old woman was referred to the pneumonology department of our hospital due to increasing productive cough and haemoptysis. During the last 30 years, she had experienced dyspnoea on exertion and increased expectoration of mucoid sputum that became purulent during infectious exacerbations, often with bloody streaked sputum. She denied fever, wheezes, chest pain, and weight loss, or any other symptom indicative of gastroesophageal reflux disease (GERD).

Her past medical history included pulmonary fibrosis diagnosed 6 years before in another country; left ovarian cancer treated with hysterosalpingo-oophorectomy 20 years before; arterial hypertension; diabetes mellitus and osteoporosis. There was no family history of any respiratory disease and she had never smoked. No history of GERD was noted in her medical records.

On physical examination, her vital signs were normal. Finger clubbing was not present. Mild inspiratory crackles at the lower third of both lung fields were revealed. Laboratory investigations were as follows: erythrocyte sedimentation rate: 15 mm/h; C-reactive protein: 1.03 mg/L with oxygen saturation at 96% on room air and arterial blood gases analysis with PaO_2_: 77.3 mmHg, PCO_2_: 41.5 mmHg, and PH: 7.45 (FiO_2_: 21%). Immunologic tests (rheumatoid factor, anti-CCP (anticyclic citrullinated peptide), C_3_, C_4_, p-ANCA, and c-ANCA) were within normal range while antinuclear antibody (ANA) levels were mildly raised (1/160) but there was no characteristic immunofluorescence pattern.

Pulmonary function tests (PFTs), conducted 1 month before her admission, revealed a forced expiratory volume in 1 sec (FEV_1_) of 1.83 L (58% predicted), a forced vital capacity (FVC) of 1.95 L (55% predicted), and FEV_1_/FVC of 105%. We were not able to perform static lung volumes measurement and diffusion test, due to the patient's inability to cooperate. Sputum results were negative for mycobacteria. Bronchoscopy was not performed because the patient did not consent.

**Figure 1:**
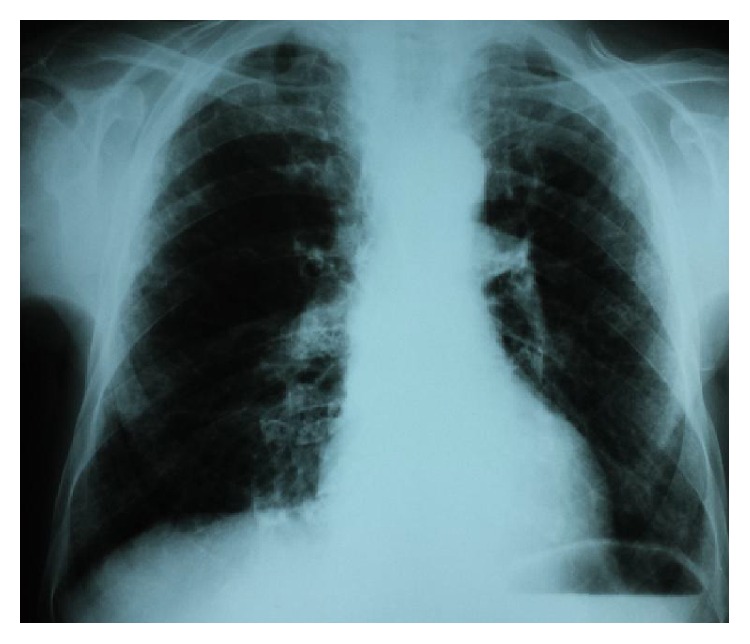
Chest X-ray conducted 24 years ago, displaying a trachea enlargement without any signs of fibrosis.

**Figure 2:**
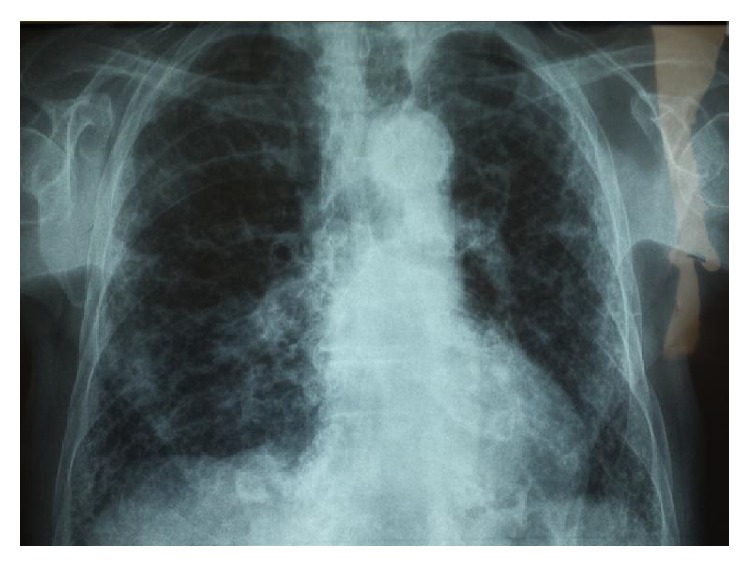
Current X-ray displaying trachea enlargement accompanied by fibrotic elements in the lungs.

**Figure 3:**
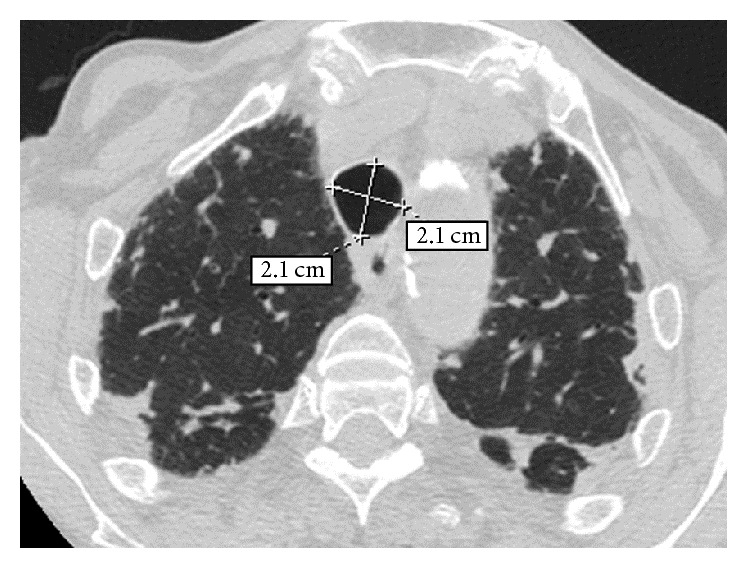
High-resolution computed tomography (HRCT) of the chest at the level of the upper lobes. An enlargement of the trachea at the thoracic inlet is observed measuring in both sagittal and axial dimensions 2.1 cm. A subpleural reticular pattern associated with focal thickening of the pleura and of the major fissures bilaterally.

**Figure 4 fig4:**
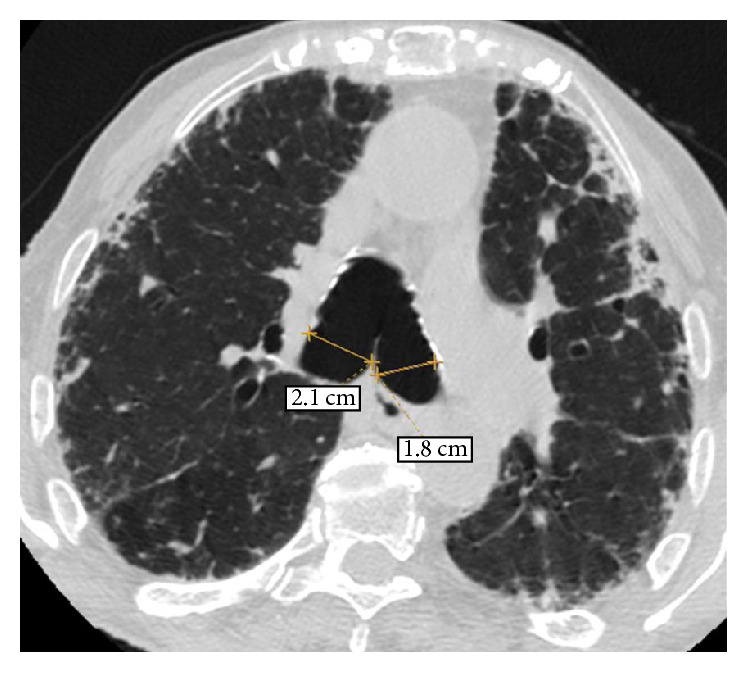
HRCT of the chest at the level of the carina. An enlargement of the right and left main bronchi is observed measuring in axial dimensions 2.1 cm and 1.8 cm, respectively. A subpleural reticular pattern associated with focal thickening of the pleura and traction bronchiectasis and bronchiolectasis is also noted.

**Figure 5 fig5:**
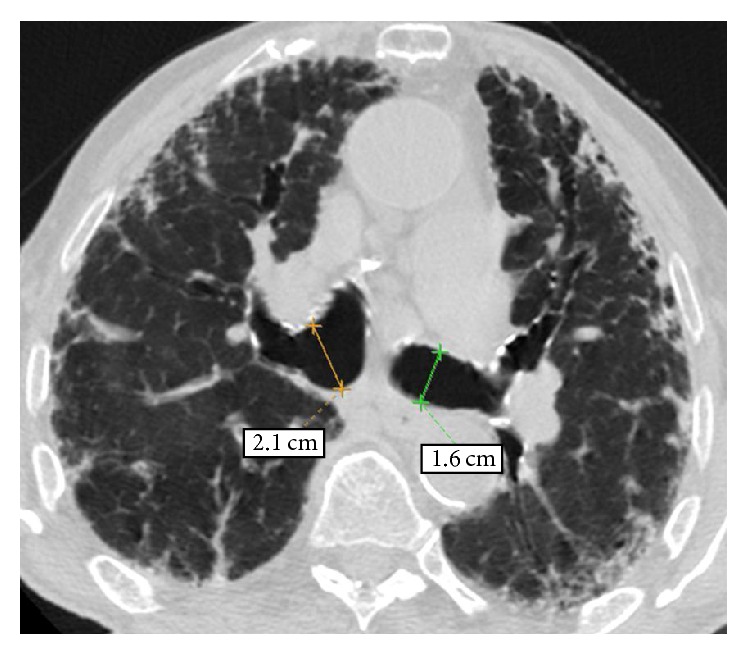
HRCT of the chest at level below carina shows dilated right and left main bronchi and undulating wall of left main bronchus, indicating bronchial diverticulosis. A subpleural reticular pattern associated with focal thickening of the pleura and traction bronchiectasis and bronchiolectasis is also noted.

**Figure 6:**
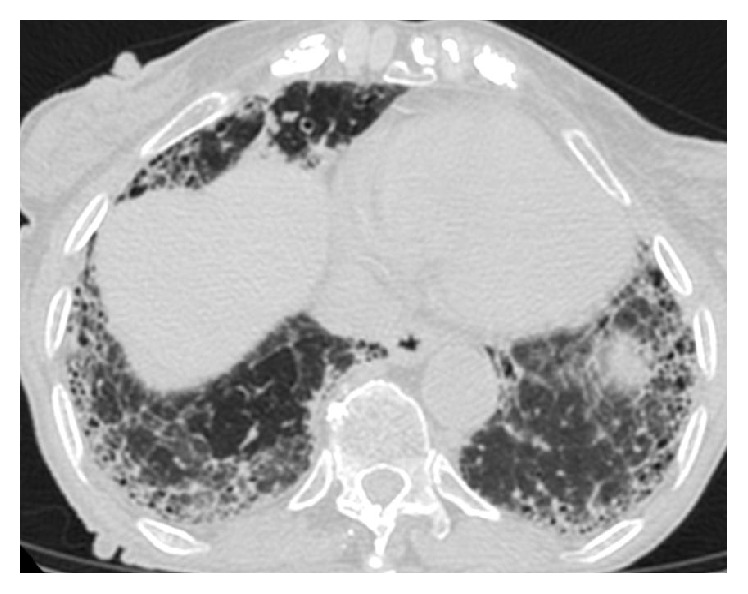
HRCT of the chest at the level of the lower lobes. An extended and coarse subpleural reticular pattern is noted associated with traction bronchiectasis and bronchiolectasis as well as with honeycombing.

An old chest radiograph, conducted 25 years ago, was available (Figure 1). This revealed widening of the trachea and main stem bronchi. These findings were confirmed by a new chest radiograph on the first day of the hospitalisation without any deterioration in terms of tracheobronchial dilatation (Figure 2). A high-resolution chest CT showed enlarged trachea and main stem bronchi (trachea with both sagittal and axial dimensions of 2.1 cm, right and left main bronchi measuring in axial dimensions 2.1 cm and 1.8 cm, resp.), extensive bronchiectasis, interstitial fibrosis of the lower lobes, and chronic pleural thickening (Figures 3 [Fig fig4] [Fig fig5]–6). The patient refused to undergo any further examination.

## 3. Discussion

This is the oldest female patient at presentation of MKS hitherto described. Although this condition is typically encountered in middle-aged men [3–5, 11–13] our patient was an elderly female, which emphasises that MKS needs to be considered in older subjects as well. Another important issue relating to the patient presented was the previous diagnosis of pulmonary fibrosis. This diagnosis rendered the recognition of MKS more difficult.

The hallmark of MKS, also known under alternative names (tracheal diverticulosis, tracheobronchiectasis, tracheocele, tracheomalacia, and tracheobronchopathia malacia), [9, 14–17] is dilatation of trachea and central bronchi with normal diameter of peripheral airways. In women, transverse and sagittal tracheal diameter must, by definition, be greater than 21 and 23 mm, respectively, [10]. There are 3 subtypes: type 1, with minimal symmetrical dilation in the trachea and main bronchi (as in the patient reported); type 2, with pronounced tracheal dilatation and diverticula; and type 3, whereby marked tracheal and bronchial dilatation extends further until the distal bronchi bilaterally [18].

Aetiology of MKS is unclear. There may be a primary defect or atrophy of elastic and smooth muscle tissue [19, 20]. MKS may also present in association with miscellaneous conditions, for example, Ehlers-Danlos syndrome, Marfan syndrome, connective tissue diseases, ataxia telangiectasia, and ankylosing spondylitis [21–24]. Diseases resulting in severe upper lobe fibrosis, such as sarcoidosis, cystic fibrosis, or diffuse pulmonary fibrosis, and airway inflammatory conditions, notably allergic bronchopulmonary aspergillosis, are also implicated in its pathogenesis [21–24]. Pulmonary fibrosis of the patient was considered idiopathic, due to the following reasons. First, serological findings were negative. Additionally any occupational exposure was excluded.

There are no pathognomonic symptoms present in MKS. Patients are usually asymptomatic while excessive sputum production may occur secondary to bronchiectasis and lower respiratory tract infection [18]. Occasional haemoptysis and dyspnoea may be seen as well [22]. In our case, the patient presented with productive cough and haemoptysis.

Its diagnosis rests on imaging studies. In the rare case of gross tracheal enlargement, chest X-rays may be diagnostic. More frequently, however, chest CT is required to reliably ascertain tracheal dimensions and to investigate any development of complications, for example, bronchiectasis [25]. At the same time, PFTs reveal a restrictive pattern.

The effect of enlarged airways on spirometry derives from the weakness of the tracheobronchial walls and hypotonia in the myoelastic elements, resulting in dynamic airway compression (expiratory collapse during forced exhalation) and dynamic restriction. The restrictive pattern in our patient is magnified from the underlying fibrosis and possibly by the associated retention of secretions. Nevertheless, these findings are not always met in MKS, because cases with normal spirometric values have been also reported [5]. Diffusion test could have been helpful; however this was not performed, due to the inability of the patient to cooperate.

Asymptomatic patients require no treatment. Therapy includes respiratory physiotherapy and antibiotics during infectious exacerbations, [26–32], while tracheal stenting is very rarely employed [2, 33].

In conclusion, this case highlights that a chest CT scan should be performed in patients reporting chronic recurrent lower respiratory tract infections to investigate underlying conditions, including MKS. Indeed, this condition, despite long-term follow-up for repeated lower respiratory infections and chronic cough, had long been left undiagnosed in our patient, until a high-resolution chest CT was performed. Our case is the oldest female patient described in literature with MKS, indicating that appropriate diagnostic workup may be required in elderly subjects as well.

## References

[1] Adani G. L., Baccarani J., Lorenzin D. (2005). Renal transplantation in a patient affected by Mounier-Kuhn syndrome. *Transplantation Proceedings*.

[2] Randak C. O., Weinberger M. (2013). A child with progressive multiple tracheal diverticulae: a variation of the Mounier-Kuhn syndrome. *Pediatric Pulmonology*.

[3] Odell D. D., Shah A., Gangadharan S. P. (2011). Airway stenting and tracheobronchoplasty improve respiratory symptoms in Mounier-Kuhn syndrome. *Chest*.

[4] Ushakumari D. S., Grewal N., Green M. (2012). Mounier-Kuhn syndrome: anesthetic experience. *Case Reports in Anesthesiology*.

[5] Krustins E., Kravale Z., Buls A. (2013). Mounier-Kuhn syndrome or congenital tracheobronchomegaly: a literature review. *Respiratory Medicine*.

[6] Enriquez G., Cadavid L., Garcés-Iñigo E. (2012). Tracheobronchomegaly following intrauterine tracheal occlusion for congenital diaphragmatic hernia. *Pediatric Radiology*.

[7] Ng J. B., Bittner E. A. (2011). Tracheobronchomegaly: a rare cause of endotracheal tube cuff leak. *Anesthesiology*.

[8] Himalstein M. R., Gallagher J. C. (1973). Tracheobronchiomegaly. *Annals of Otology, Rhinology & Laryngology*.

[9] Mounier-Kuhn P. (1932). Dilatation de la trachee: constatations, radiographiques et bronchoscopies. *Lyon Medical*.

[10] Fraser R. S., Pare P. D., Muller N. L., Colman N. (1999). Bronchiectasis and other bronchial abnormalities. *Diagnosis of Diseases of Chest*.

[11] Jaiswal A. K., Munjal S., Singla R., Jain V., Behera D. (2012). A 46-year-old man with tracheomegaly, tracheal diverticulosis, and bronchiectasis: Mounier-Kuhn syndrome. *Lung India*.

[12] Arroyo-Cózar M., Ruiz-García M., Merlos E. M., Vielba D., Macías E. (2012). Case report: respiratory infection due to alcaligenes xylosoxidans in a patient with mounier-kuhn syndrome. *Revista Chilena de Infectologia*.

[13] Bastos A. D. L., Brito I. L. A. (2011). Mounier-kuhn syndrome: radiological findings and clinical presentation. *Radiologia Brasileira*.

[14] Czyhlarz E. R. (1897). Über Pulsionsdivertikel der Trachea mit Bemerkungen über das Verhalten der elastischen Fasern an normalen Tracheen und Bronchien. *Zentralblatt für Allgemeine Pathologie und Pathologische Anatomie*.

[15] Bateson E. M., Woo-Ming M. (1973). Tracheo-bronchomegaly. *Clinical Radiology*.

[16] Gay S., Dee P. (1984). Tracheobronchomegaly-the Mounier-Kuhn syndrome. *British Journal of Radiology*.

[17] Engle W. A., Cohen M. D., McAlister W. H., Griscom N. T. (1987). Neonatal tracheobronchomegaly. *American Journal of Perinatology*.

[18] Schwartz M., Rossoff L. (1994). Tracheobronchomegaly. *Chest*.

[19] Katz I., Levine M., Hermam P. (1962). Tracheobronchomegaly (Mounier-Kuhn Syndrome): CT diagnosis. *American Journal of Roentgenology*.

[20] Spencer H., Spencer H. (1985). Congenital abnormalities of the lung: congenital tracheobronchomegaly. *Pathology of the Lung*.

[21] Blake M. A., Clarke P. D., Fenlon H. M. (1999). Thoracic case of the day: Mounier-Kuhn syndrome (tracheobronchomegaly). *American Journal of Roentgenology*.

[22] Van Schoor J., Joos G., Pauwels R. (1991). Tracheobronchomegaly: the Mounier-Kuhn syndrome: report of two cases and review of the literature. *European Respiratory Journal*.

[23] Sane A. C., Effmann E. L., Brown S. D. (1992). The Mounier-Kuhn syndrome in a patient with the Kenny-Caffey syndrome. *Chest*.

[24] Woodring J. H., Barrett P. A., Rehm S. R., Nurenberg P. (1989). Acquired tracheomegaly in adults as a complication of diffuse pulmonary fibrosis. *American Journal of Roentgenology*.

[25] Lakshminarayana P. H., Woodske M. E. (2012). Mounier-Kuhn syndrome: imaging in recurrent pulmonary infections. *American Journal of Respiratory and Critical Care Medicine*.

[26] Noori F., Abduljawad S., Suffin D. M. (2010). Mounier-Kuhn syndrome: a case report. *Lung*.

[27] Ip J. J., Hui P. K., Lam S. H., Lam W. W., Chau M. T. (2013). Mounier-Kuhn syndrome: an unusual underlying cause for chronic coughs and recurrent pneumonias. *Hong Kong Medical Journal*.

[28] Khasawneh F. A., Jou-Tindo A. J. (2013). A 30-year-old woman with recurrent lower respiratory tract infections. *Chest*.

[29] Pacheco M. C., Sancho-Chust J. N., Chiner E. (2010). Mounier-Kuhn syndrome diagnosed in an adult. *Archivos de Bronconeumologia*.

[30] Dalar L., Eryüksel E., Koşar F. (2012). Central airway obstruction due to malignant fibrous histiocytoma metastasis in a case with Mounier-Kuhn syndrome. *Tuberkuloz ve Toraks*.

[31] Kent B. D., Sulaiman I., Akasheh N. B., Nadarajan P., Moloney E., Lane S. J. (2011). An unusual cause of spontaneous pneumothorax: the Mounier-Kuhn syndrome. *Irish Medical Journal*.

[32] Lyons O. D., Gilmartin J. J. (2014). A grossly abnormal trachea- severe tracheal diverticulosis and Mounier-Kuhn syndrome. *Irish Medical Journal*.

[33] Dutau H., Maldonado F., Breen D. P., Colchen A. (2011). Endoscopic successful management of tracheobronchomalacia with laser: apropos of a Mounier-Kuhn syndrome. *European Journal of Cardio-Thoracic Surgery*.

